# A Spontaneous Complexation–Exfoliation Strategy for a Flexible Anode Towards Superior Durable and Ultrafast Lithium-Ion Batteries

**DOI:** 10.3390/molecules30010133

**Published:** 2024-12-31

**Authors:** Heying Chu, Jingchuan Zhang, Pengsen Zhao, Yong Li, Zhaoxia Liu, Hongzhou Zhang

**Affiliations:** 1 College of Mechanical and Electronic Engineering, Tarim University, Alar 843300, China; chuheying@taru.edu.cn (H.C.); zhangjingchuan@taru.edu.cn (J.Z.); zpstaru@163.com (P.Z.); deyuzhijia@163.com (Y.L.); 2 College of Mechanical and Electronic Engineering, Wuhan Donghu University, Wuhan 430017, China

**Keywords:** flexible electrode, free-standing, spontaneous complexation and exfoliation, layer-embedded structure, high rate, lithium-ion batteries

## Abstract

Transition metal oxides are considered promising anode materials for high performance flexible electrodes due to their abundant reserves and excellent specific capacity. However, their inherent low conductivity, large volume effect, and poor cycling performance limit their applications. Herein, we report a novel “spontaneous complexation and exfoliation” strategy for the fabrication of flexible MnO NCs@rGO thin-film electrodes, which overcomes the aforementioned drawbacks and pushes the mechanical flexibility and lithium-ion (Li^+^) storage performance to a higher level. The combination of large-area few-layer reduced graphene oxide (rGO) films and ultrafine MnO nanocrystals (MnO NCs) provides a high density of electrochemical active sites. Notably, the layer-by-layer embedded structure not only enables the MnO NCs@rGO electrodes to withstand various mechanical deformations but also produces a strong synergistic effect of enhanced reaction kinetics by providing an enlarged electrode/electrolyte contact area and reduced electron/ion transport resistance. The elaborately designed flexible MnO NCs@rGO anode provides a specific capacity of about 1220 mAh g^−1^ over 1000 cycles, remarkable high-rate capacity (50.0 A g^−1^), and exceptional cycling stability. Finally, the assembled flexible lithium-ion full cells achieve zero capacity loss during repeated large-angle bending, demonstrating immense potential as a high-performance flexible energy storage device. This work provides valuable insights into unique structural designs for durable and ultra-fast lithium ion batteries.

## 1. Introduction

Rapid advances in electronics have contributed significantly to the development of portable and wearable electronic devices that require high-performance batteries to operate [1,2]. Currently, the technology landscape is accelerating the shift towards flexibility and health-centric designs in electronic products [3]. The advent of bendable displays and chips in electronic devices has paved the way for innovative, flexible applications [4]. To achieve this vision, however, a crucial enabler is the cooperation of flexible energy storage devices. Lithium-ion batteries (LIBs) are currently widely used in small and medium-sized portable electronic products due to a number of advantageous characteristics, including high energy density, high output voltage, long cycle lifespan, and environmental friendliness [5]. However, there are still significant challenges to overcome in the application of flexible devices, particularly the necessity to combine electrode materials with favorable mechanical durability, minimal weight, elevated specific capacity, extended cycle life, and optimal safety [6].

Commercial LIBs are currently characterized by a substantial degree of bulk and weight [7,8], which can be attributed to the utilization of a considerable number of collectors, binders, and conductive agents. Compared to conventional electrodes, the self-supporting flexible electrodes eliminate the necessity for these inactive components. This increases the specific capacitance, reduces the cost and weight of the electrode material, and provides the opportunity for improved flexibility and processability [9]. Graphite, a traditional anode material, is inadequate for meeting the requirements of future high-specific-energy and high-capacity batteries due to its low theoretical specific capacity (372 mAh g⁻^1^) and poor rate performance [10]. Other intercalated anode materials (such as Li_4_Ti_5_O_12_ and TiO_2_), known as “volumetric zero strain” materials, exhibit extremely long cyclic stability, but are limited by poor conductivity and high reaction potential [11,12,13]. Therefore, it is imperative to design and fabricate a new generation of flexible electrodes with higher reversible capacity, high-rate performance, long life, and low cost. Transition metal oxides, including CoO [14], Co_3_O_4_ [15,16], Fe_2_O_3_ [17], Fe_3_O_4_ [18,19], SnO_2_ [20,21,22], NiO [23], MnO [24,25,26], and MoO_2_ [27,28], etc., which exhibit high theoretical specific capacities, have emerged as potential candidates for high-performance anode materials. Among these, MnO nanocrystals have received increasing attention due to their numerous favorable properties, including relatively lower electrode potentials (1.032 V vs. Li^+^/Li), high theoretical capacity, abundant reserves, and environmental compatibility [25]. However, the inherent low conductivity and the huge volume expansion of MnO electrodes during electrochemical processes result in structural collapse, rapid capacity decay, and difficulty withstanding high current charging. Furthermore, the discharge products, namely metallic Mn and Li_2_O particles, are prone to agglomeration and precipitation. Researchers have endeavored to address these issues using a variety of means [24]. Of these, nanosizing and carbon-coating strategies are considered to be the most effective [29,30,31]. Nanosized particles can increase the electrochemical active sites and shorten the Li^+^ transport distance, while carbon-coating can improve the conductivity, prevent the agglomeration of metal particles, and serve as a bulk strain relief layer to maintain the stability of the electrode material structure. However, the challenge remains in regards to achieving an ultrafine nanoparticle with a perfect carbon coating that exhibits optimal performance.

Herein, an “autonomous chelation and exfoliation” film-forming method was developed at room temperature, resulting in a layer-by-layer embedded flexible self-supported MnO NCs@rGO nanocomposite film after subsequent sintering. Specifically, the ultrafine MnO NCs are uniformly dispersed and confined within the rGO films, thereby forming a large-area three-dimensional ordered conductive network. The elaborately prepared MnO NCs@rGO film electrode perfectly inherits the advantages of rGO, including a thin layer, good conductivity, low weight, large specific surface area, and robust mechanical properties. Additionally, it effectively overcomes the inherent limitations of MnO, such as low conductivity, a huge volume effect, and rapid capacity decline. As an anode material for LIBs, the MnO NCs@rGO film displays ultra-high specific capacity, excellent cycling stability (1220 mAh g^−1^ over 1000 cycles), and exceptional rate performance (336.6 mAh g^−1^ at 50.0 A g^−1^). Ultimately, the assembled flexible LIBs’ full cells (MnO NCs@rGO//LiCoO_2_) demonstrate remarkable flexibility and zero-diminished electrochemical stability. This study offers a valuable methodology for the development of ultra-light, flexible, and high-performance electrode materials for LIBs.

## 2. Results

### 2.1. Synthesis and Structural Characterization

**Figure 1:**
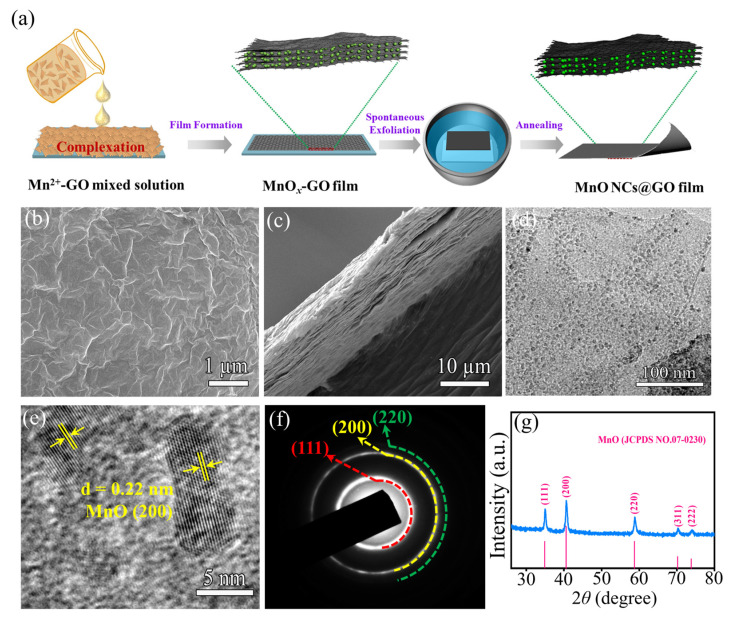
(**a**) Schematic diagram of the synthesis process of flexible MnO NCs@rGO thin films. (**b**) Surface and (**c**) cross-section SEM images, (**d**) TEM image, (**e**) HRTEM image, (**f**) corresponding SAED pattern, and (**g**) XRD pattern of flexible MnO NCs@rGO film.

Figure 1a illustrates the fabrication process of MnO NCs@rGO flexible films. A solution comprising manganese salts and graphene oxide (GO) is automatically chelated, deposited, and forms a dense film on a glass substrate under the reducing stimulus of HI acid [32]. Subsequently, the film is automatically stripped from the glass plate by infiltration into water. Following the high-temperature reduction process, the MnO NCs@rGO film is obtained. The delicate fabrication process of MnO NCs@rGO film can be attributed to the following aspects: firstly, the positive Mn^2+^ bond spontaneously interacts (interactions between -O···M and -COO···M) with GO, containing abundant oxygen-containing functional groups (-OH, -COOH, -O-) and electronegativity, and ultimately homogeneously anchors onto the surface of the GO nanosheets. Subsequently, the strong complexation and reduction property of the I^−^ ions promotes the colloidal association of GO, forming a large, dense rGO composite film. Finally, the composite films with free oxidized functional groups exhibit notable hydrophobicity, thereby enabling the spontaneous exfoliation from water (Figure S1, with an area of about 33 cm × 15 cm).

Figure S2 shows a piece of MnO NCs@rGO film material with an area of 19 cm × 8 cm. It is evident that the composite film remains intact and dense, exhibiting notable flexibility and the ability to be bent randomly at large angles. In detail, the surface of the MnO NCs@rGO flexible film is rich and uniformly distributed with wrinkles formed by the interconnection of ultrathin rGO layers (as shown in Figure 1b). The smooth surface indicates that the MnO NCs exhibit an ultrafine particle size and are firmly embedded in the rGO interlayers. Furthermore, the cross-sectional scanning electron microscope (SEM) image (Figure 1c) clearly demonstrates that the thickness of the MnO NCs@rGO film is approximately 20 µm and consists of a uniform stacking of ultrathin rGO sheets, with a certain degree of interlayer spacing. This layer-embedded structure avoids MnO agglomeration and precipitation, enhances electrolyte wetting, facilitates rapid electron transport, inhibits the volume expansion of the active material during charging and discharging, and improves the mechanical stability and electrochemical lifetime. In contrast, the manganese oxide that was not protected by rGO exhibited a significant agglomeration phenomenon (as shown in Figure S3).

In order to gain insight into the existence form, distribution, and crystal structure of MnO NCs in the ultrathin layer structure of rGO, transmission electron microscopy (TEM), selected area electron diffraction (SAED), and X-ray diffraction (XRD) were employed. The TEM test results, as shown in Figure 1d and Figure S4, demonstrate that the ultrafine MnO nanocrystals (with particle sizes of approximately 5~10 nm) are uniformly distributed on the surfaces of the ultrathin rGO layers. Moreover, the monodisperse MnO NCs display a discernible distance between them, indicating that the oxygen-containing functional groups in the GO effectively inhibit the agglomeration of the MnO NCs, even during the high-temperature reduction process. An appropriate spacing can also effectively prevent particle agglomeration during the charge/discharge cycling process, thereby enhancing the cycling stability of the material. Furthermore, the high-resolution TEM (HRTEM) images show an obvious lattice spacing of 0.318 nm (Figure 1e), corresponding to the (200) crystal plane of the cubic phase of MnO (JCPDS No. 07-0230) [33]. The corresponding SAED pattern in Figure 1f also identifies several crystal planes of MnO, including (200), (220), and (111). Subsequent XRD results (as shown in Figure 1g) display a series of diffraction peaks at 34.9, 40.5, 58.7, 70.2, and 73.8° corresponding to the (111), (200), (220), (311), and (222) crystal planes, which are consistent with the results for cubic MnO [33]. The broad diffraction peaks also suggest that the MnO nanoparticles are relatively small in size.

**Figure 2:**
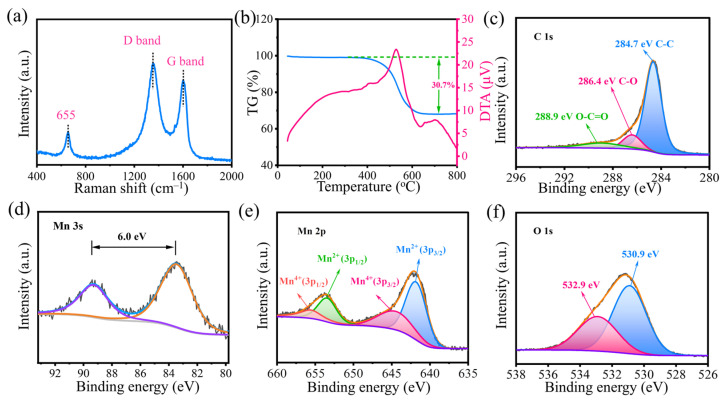
Material characterization of the MnO NCs@rGO film. (**a**) Raman spectra; (**b**) TG-DTA curve obtained at a heating rate of 10 °C min^−1^ in flowing air. High-resolution XPS spectra of (**c**) C 1s, (**d**) Mn 3s, (**e**) Mn 2p, and (**f**) O 1s.

A systematic characterization was conducted using Raman, thermogravimetric-differential thermal analysis (TG-DTA), and X-ray photoelectron spectroscopy (XPS) tests to further clarify the state and content of MnO, as well as the presence of valence states of each element in the flexible MnO NCs@rGO thin films. Two distinct characteristic peaks located at 1333 and 1578 cm⁻^1^, observed in the Raman results, can assigned to the D- and G-bands of carbon [34], respectively (Figure 2a). The corresponding I_D_/I_G_ ratio is 1.17, indicating that the MnO NCs@rGO composite displays a higher density of defects and active sites. Furthermore, the contents of carbon and MnO in the MnO NCs@rGO composite were determined by the TG-DTA in air. As illustrated in Figure 2b, a pronounced decline in mass is observed in the 400–650 °C range, followed by a gradual increase in the 650–800 °C range, which is attributed to the oxidation reactions of MnO and C, respectively, as follows: (1) C + O_2_ → CO_2_↑; (2) 2MnO + O_2_ → 2MnO_2_. The DTA curves display a pronounced exothermic peak at about 520 °C, indicative of the combustion of rGO, while a less intense exothermic peak at about 600 °C is suggestive of the oxidation of MnO. In light of the aforementioned results, the contents of MnO and rGO in MnO NCs@rGO are calculated to be 58.1% and 41.9%, respectively. In addition, the characteristic signals of Mn (2p_1/2_, 2p_3/2_, and 3s), O (1s), and C (1s) were identified in the full XPS spectrum (Figure S5). In Figure 2c, the high-resolution spectrum of the C 1s peaks can be fitted to four peaks. The strongest peak at 284.4 eV is attributed to graphitic carbon in rGO, while the other three weaker peaks are ascribed to the C-O bond (286.2 eV), the C=O bond (287.9 eV), and the O-C=O bond (289.0 eV), respectively [35]. A spin-energy separation of 6.0 eV for the Mn 3s doublet (as shown in Figure 2d) indicates that the Mn in the MnO NCs@rGO has a charge state of ~2. The Mn 2p spectrum, as shown in Figure 2e, can be divided into two peaks, located at 642.1 and 653.5 eV, which correspond to Mn 2p_3/2_ and Mn 2p_1/2_, respectively. Furthermore, the two peaks observed at 530.9 and 532.9 eV in the O 1s spectrum are ascribed to the Mn–O bonds and the surface oxidation layer of the rGO matrix (C–O–C) (Figure 2f) [36].

### 2.2. Electrochemical Properties

**Figure 3:**
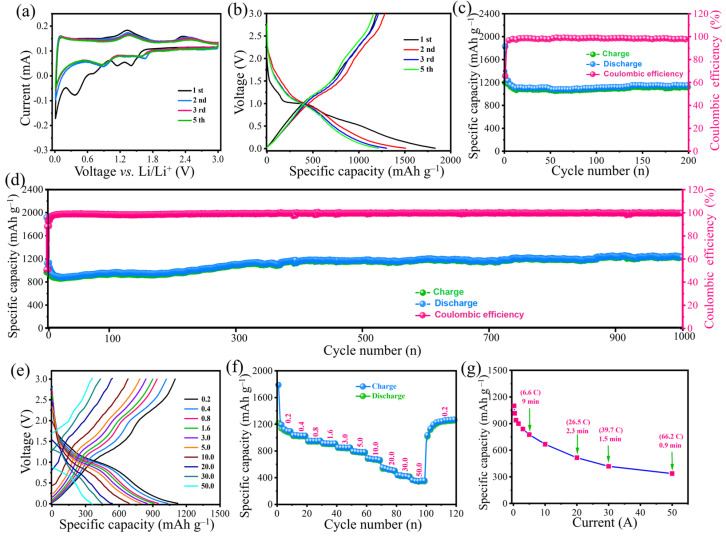
Electrochemical performance of the flexible MnO NCs@rGO film electrode: (**a**) CV curves 0.1 mV s^−1^; (**b**) galvanostatic charge/discharge profiles and cycling performance at (**c**) 200 mA g^−1^ and (**d**) 500 mA g^−1^; (**e**) galvanostatic charge/discharge profiles, (**f**) rate capability, and (**g**) corresponding relationship between current density and charge/discharge time at different current densities from 0.2 A to 50 A g^−1^.

A series of coin-type half-cells were assembled and coupled with Li metal as the counter electrode to evaluate the electrochemical behavior of the MnO NCs@rGO film electrode. Figures S6 and S7 present the crystalline phase and thin-layered structure of rGO, as well as optical photographs of the flexible self-supported structure of the MnO NCs@rGO film electrode. Figure 3a depicts the initial five cyclic voltammetry (CV) profiles of the MnO NCs@rGO film electrode within the voltage range of 0.01–3.0 V (vs. Li/Li^+^) at a scan rate of 0.1 mV s^−1^. In the initial cathodic loop, two cathodic peaks are observed at 0.68 and 0.26 V, which can be attributed to the reduction of Mn^2+^ to Mn^0^, accompanied by the formation of Li_2_O (MnO + 2Li^+^ + 2e^−^ → Mn + Li_2_O) and the formation of the solid electrolyte interphase (SEI) layer [37,38]. Two additional peaks, situated at 1.41 and 1.14 V, may be due to the reduction of residual high-valent manganese oxide (MnO*_x_*, 2 < *x* < 3) on the surface of the rGO and the passivation reaction of the collector [38]. During the oxidative stage, two peaks at 1.33 V and 2.34 V are indicative of the gradual oxidative reaction of Mn^0^, with a minor proportion of Mn^2+^ undergoing transformation to the valence of Mn*^x^*^+^ at elevated potentials [39]. Following the microstructural recombination of the electrode materials after the initial lithiation process, the reduction/oxidation peaks corresponding to MnO (located at 0.88/1.33 V) in the 2nd to 5th cycles were confirmed. In parallel, the reduction/oxidation peaks of Mn*^x^*^+^ were gradually weakened as a result of the microstructural reorganization of the electrode material. As illustrated in Figure 3b, the voltage plateau of the galvanostatic charge/discharge (GCD) curves coincides with the position of the redox peak observed in the CV results. The initial discharge and charge capacities of the MnO NCs@rGO film electrode were determined to be 1830 and 1160 mAh g^−1^, respectively, with an initial Coulombic efficiency of 65% (based on the total mass of the MnO NCs@rGO film electrode). The irreversible capacity loss observed during the initial cycles was primarily attributed to the consumption of active lithium ions involved in the formation of the solid electrolyte interface (SEI) film. Subsequently, the Coulomb efficiency gradually increased, approaching 100% after three cycles, while the discharge and charge curves were essentially coincident. In terms of cycling stability, the MnO NCs@rGO film anode maintains a specific capacity of 1121 mAh g^−1^ after 200 cycles at a low current density of 0.2 A g^−1^, with a capacity retention rate of about 96.8% (Figure 3c). In comparison, the MnO electrode demonstrated a tendency towards rapid capacity decline, while the pure rGO paper exhibited a considerably lower capacity than that of the MnO NCs@rGO film anode (Figures S8 and S9). Surprisingly, the specific capacity of the MnO NCs@rGO film anode remained stable after 1000 charge/discharge cycles at 500 mA g^−1^ (as shown in Figure 3d). Moreover, it showed a slight increase, which has been documented for numerous transition metal oxide anode materials [39,40]. This phenomenon can be attributed to the nanosizing of active materials, the expansion of the specific surface area, and the enhancement of electrochemical sites during high-current charging and discharging [41]. In contrast, the two opposing electrodes (MnO and rGO anodes) showed a rapid decrease in capacitance at the outset of the electrochemical reaction and maintained a very low capacitance thereafter, which can be attributed to structural instability (Figure S10).

Furthermore, Figure 3e,f illustrates the constant GCD and rate performance curves at different current densities (0.2−50.0 A g^−1^). Clearly, the voltage plateaus of the GCD curves are obvious, and with increasing current density, the plateaus regularly weaken, but these plateau positions remain relatively stable, which is attributed to the superconductivity reducing the electrochemical polarization. Figure 3f shows the excellent rate performance of the MnO NCs@rGO film electrode, exhibiting charge-specific capacities of 1100, 1015, 938.5, 899, 838.7, 775.4, 667, 515, 418.5, and 336.6 mAh g^−1^ at current densities of 0.2, 0.4, 0.8, 1.6, 3.0, 5.0, 10.0, 20.0, 30.0, and 50.0 A g^−1^, respectively, which are much higher than those of the MnO, rGO electrodes (Figure S11) and electrode materials, based on those reported for MnO [6,24,25,26,27,37,40]. Surprisingly, even at an ultra-high current density of 50.0 A g^−1^ (equivalent to 66.2 C), the MnO NCs@rGO film electrodes demonstrate a reversible capacity of 336.6 mAh g^−1^, which corresponds to the completion of a charge/discharge time of only 0.9 min (Figure 3g). The ultra-high current tolerance and corresponding ultra-high capacity indicate that the MnO NCs@rGO anodes possess exceptionally robust structural stability. The unrivalled rate performance and structural stability of the MnO NCs@rGO film anode are significantly superior to those reported for MnO*_x_*-based electrodes (Table S1). As anticipated, the favorable cycling stability and excellent rate performance indicate a promising potential application in future high-specific-energy lithium-ion secondary batteries.

### 2.3. Electrochemical Reaction Mechanism

Furthermore, ex situ XRD tests were employed to investigate the detailed lithium-ion storage mechanism of the MnO NCs@rGO film anode, as shown in Figure S12. A diffraction peak is consistently present at 43.3°, attributed to the copper current collector (JCPDS No. 99-0034). The initial characteristic peaks at around 34.9 and 40.5 correspond to the (111) and (200) crystal planes, respectively, of the cubic phase of MnO (JCPDS No. 07-0230). Obviously, the diffraction peaks of MnO gradually weakened and disappeared at 0.38 V during discharge and were regenerated during subsequent charging. Meanwhile, the diffraction peaks corresponding to the rGO (002) crystal surface (located at 21.2°) gradually shifted to a smaller angle during the discharge process, confirming the expansion of the interlayer spacing, which can be attributed to the intercalation and adsorption of lithium ions within the interlayers. On the contrary, the (002) peak recovers to 21.2° after charging back to 3.0 V, indicating the outstanding structural stability and electrochemical reversibility of the MnO NCs@rGO film electrode. Combined with the CV test results, it can be concluded that MnO in the MnO NCs@rGO electrodes follows a stepwise conversion–reversion conversion reaction mechanism, while rGO undergoes an intercalation–adsorption reaction process.

### 2.4. Kinetic Analysis

**Figure 4:**
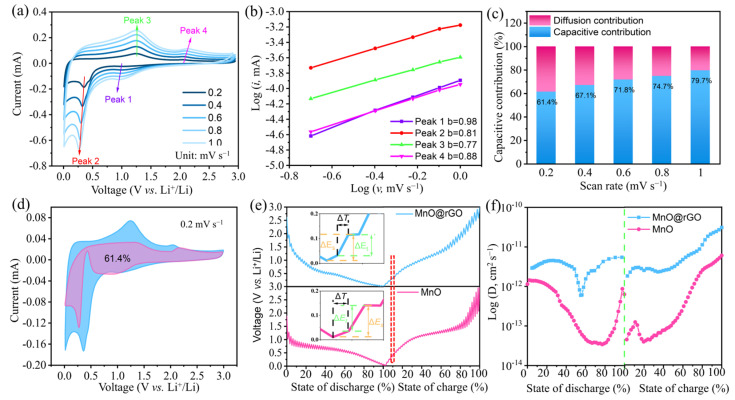
Dynamic behavior analysis of the flexible MnO NCs@rGO film electrode: (**a**) CV curves at various scan rates, (**b**) the relationship between log *i* and log *v*, (**c**,**d**) capacity contribution ratios of capacitive-controlled behavior of the flexible MnO NCs@rGO film electrode. (**e**) GITT curves and (**f**) corresponding D_Li+_ values for the MnO NCs@rGO film and MnO electrodes.

To gain further insight into the underlying mechanisms for the excellent rate performance of the MnO NCs@rGO film electrodes, a series of cyclic voltammetry tests and galvanostatic intermittent titration technique (GITT) tests were performed at different scan speeds. The corresponding Li-ion storage behavior and Li^+^ diffusion coefficient (D_Li+_) was then summarized. In the CV curves (as shown in Figure 4a), the reduction and oxidation peaks of the MnO NCs@rGO film electrode shifted in opposite directions with an increasing scan rate (from 0.2 to 1.0 mV s^−1^). The calculated *b*-values of the linear fits corresponding to the reduction (peak 1, peak 2) and oxidation (peak 3, peak 4) peaks are 0.98, 0.81, 0.77, and 0.88, respectively (Figure 4b), suggesting that the MnO NCs@rGO film electrodes follow the pseudo-capacitance-dominated “capacitor-battery” dual-type lithium storage mechanism [42,43]. Moreover, the capacitance contributions of the MnO NCs@rGO film electrodes were calculated to be 61.4%, 67.1%, 71.8%, 74.7%, and 79.7% for the scan rates of 0.2, 0.4, 0.6, 0.8, and 1.0 mV s^−1^, respectively (Figure 4c,d). The high pseudocapacitance is mainly due to the abundant active surface area afforded by the layered rGO and the ultrafine MnO particles, as well as the presence of highly active electrochemical reaction sites provided by the MnO NCs. Subsequently, the diffusion coefficient of Li^+^ in the MnO NCs@rGO film electrode was evaluated after 20 cycles using the GITT, applying a series of pulse currents at 0.02 A g^−1^ for 30 min (τ) and allowing it to remain stationary for 30 min. As depicted in Figure 4e, both the MnO NCs@rGO film and the MnO electrodes exhibit similar potential distribution trends throughout the entire discharging/charging processes, indicating the same Li-ion storage mechanism. The calculated D_Li+_ of MnO NCs@rGO was found to be 5.98 × 10^−12^–2.97 × 10^−11^ cm^2^ s^−1^, which significantly higher than that of MnO (3.22 × 10^−12^–5.53 × 10^−12^ cm^2^ s^−12^) (Figure 4f). The aforementioned kinetic analyses have demonstrated that the MnO NCs@rGO film electrode with a layer-embedded structure can significantly accelerate Li^+^ transport, thus resulting in its superior rate performance and lithium storage kinetics. In summary, the exceptional electrochemical performance of MnO NCs@rGO film electrodes can be attributed to the ingenious structural design, as illustrated below: (1) the large-area rGO nanosheets and ultrafine MnO nanoparticles provide abundant electrochemical active sites; (2) the rGO-based film possesses an abundant fold-like structure to provide a sufficient attachment interface for the MnO, also providing a volume-release space for repeated charge/discharge processes; (3) the layer-by-layer embedded three-dimensional structure promotes electrolyte infiltration and storage and shortens the distance for ion transport; (4) most importantly, the strong synergistic effect between rGO nanosheets and MnO NCs significantly improves the ability of the composite electrode to withstand mechanical deformation, expands the electrode/electrolyte contact area, reduces the electron/ion transport resistance, and improves the reaction kinetics.

### 2.5. Flexible Full-Cell Assembly and Electrochemical Properties

**Figure 5:**
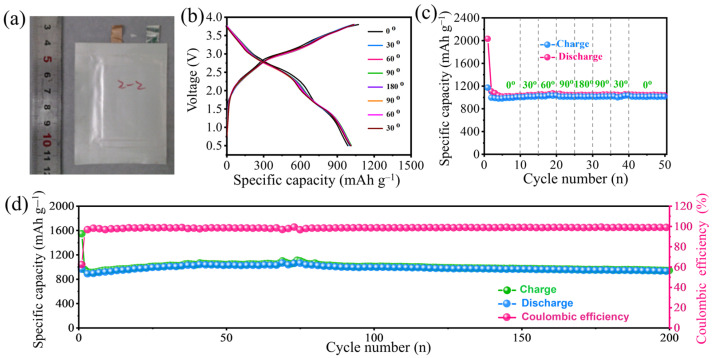
(**a**) Photograph, (**b**) GCD curves, and (**c**) corresponding cycling performance of the flexible full cell with different bend statuses. (**d**) Long-term cycling performance and Coulombic efficiency of the full cell at a current density of 500 mA g^−1^.

In light of the aforementioned exceptional electrochemical properties, remarkable flexibility, and robust mechanical properties, the MnO NCs@rGO film electrode has essentially fulfilled the criteria for high-performance electrode materials in flexible LIBs. To assess the practicality of the MnO NCs@rGO film anode when coupled with the LiCoO_2_ cathode and an aluminum-coated plastic film shell, a flexible lithium-ion full cell (MnO NCs@rGO//LiCoO_2_) was constructed after vacuum encapsulation. As illustrated in Figure 5a, the size of the flexible full cell is 5.5 ×7.5 × 0.1 cm. Long strips of aluminum and copper foil are employed to facilitate contact with a limited portion of the electrode materials and are used as battery tabs for the cathode and anode of the full cell. Initially, a low current density of 20 mA^−1^ was applied during the initial galvanostatic charge/discharge process, which is considered as the cell formation process. In order to optimize the cell capacity, the voltage range of the full cells was set to 0.5–3.9 V. Figure 5b,c shows the GCD curves and cycle performance results of the MnO NCs@rGO//LiCoO_2_ full cell at different bending angles under 200 mA g^−1^, with each bending angle performed for 10 cycles. It can be observed that the initial charge-/discharge-specific capacities of the MnO NCs@rGO//LiCoO_2_ full cell were 2015 and 1195 mAh g^−1^, respectively, with a Coulombic efficiency of approximately 60% (Figure 5b). Following the bending of the flexible full cell to 30 °, 60 °, 90 °, and 180 ° and subsequent reversible restoration to 90 °, 60 °, 30 °, and 0 °, respectively, the reversible capacity remained almost constant (Figure 5c), confirming the excellent flexibility and stable electrochemical stability of the MnO NCs@rGO//LiCoO_2_ full cell. The additional stability test, as illustrated in Figure 5d, demonstrated that the charge-specific capacity of the MnO NCs@rGO//LiCoO_2_ full cell remained at approximately 941.8 mAh g^−1^ over 200 cycles at 500 mA g^−1^, with a capacity retention rate of nearly 100%. It is noteworthy that the full cell exhibited a consistently high Coulombic efficiency, exceeding 99% throughout the cycling period. In order to assess the practicality of the full cell, it was bent and connected to an array of about 100 LEDs, which were successfully illuminated (Figure S13). These favorable results in terms of bending performance and electrochemical stability indicate that the MnO NCs@rGO//LiCoO_2_ full cells have the potential for commercialization in flexible and wearable optoelectronic devices.

## 3. Conclusions

In summary, we have successfully synthesized a layer-embedded structured, flexible MnO NCs@rGO thin-film electrode using a novel “spontaneous complexation–exfoliation” strategy. This method utilizes the nucleophilicity of GO surface functional groups and the surface hydrophobicity of rGO to achieve the spontaneous complexation–exfoliation process, thereby indicating its feasibility for simple and large-scale application. Benefiting from the synergistic effect of the thin film, its flexibility, the strong mechanical properties of rGO, and the numerous active sites and high capacity of ultrafine MnO NCs, the flexible MnO NCs@rGO anodes completely overcame the inherent drawbacks of MnO electrodes, including low conductivity, huge volume effect, and rapid capacity degradation. Consequently, the lithium storage capacity and fast charge/discharge capability have reached new heights. Most impressively, the assembled soft-pack lithium-ion battery can withstand full-angle bending without any capacity loss. These results demonstrate the rationality and broad application prospects of the layered embedded structure.

## Data Availability

Dataset available upon request from the authors.

## Supplementary Materials

The following supporting information can be downloaded at: https://www.mdpi.com/article/10.3390/molecules30010133/s1, Figure S1: Photograph of the MnOx-rGO intermediate and flexible MnO NCs@rGO film.; Figure S2: SEM image and XRD patterns of MnO; Figure S3: HR-TEM image of the flexible MnO NCs@rGO film; Figure S4: XRD pattern and SEM image of rGO; Figure S5: Photograph of the flexible free-standing MnO NCs@rGO electrode; Figure S6: GCD profiles and cycling performance of the MnO anode at 0.2 A g^−1^; Figure S7: GCD profiles and cycling performance of the rGO anode at 0.2 A g^−1^; Figure S8: Cycling performance of the MnO and rGO anode at 0.5 A g^−1^; Figure S9: Rate performance of the MnO and rGO anode at different current densities from 0.2 A to 50.0 A g^−1^; Figure S10: Photograph of the flexible MnO NCs@rGO//LiCoO_2_ full cell, powering an array of 100 LEDs. Refs. [44,45,46] are cited in the Supplementary Materials.
