# Supplementary material for: A Spontaneous Complexation–Exfoliation Strategy for a Flexible Anode Towards Superior Durable and Ultrafast Lithium-Ion Batteries
Source: Molecules. 2024 Dec 31;30(1):133. doi: 10.3390/molecules30010133 (PMC11721905; doi:10.3390/molecules30010133)
Supplement: Supplementary file 1 [file molecules-30-00133-s001.zip › molecules-3349868-supplementary.pdf]

A Spontaneous Complexation–Exfoliation Strategy for a Flexible Anode Towards Superior Durable and Ultrafast Lithium-Ion Batteries

Heying Chu [1], Jingchuan Zhang [1], Pengsen Zhao [1], Yong Li [1], Zhaoxia Liu [2,*] and Hongzhou Zhang [1,*]

[1] College of Mechanical and Electronic Engineering, Tarim University, Alar 843300, China; chuheying@taru.edu.cn (H.C.); zhangjingchuan@taru.edu.cn (J.Z.); zpstaru@163.com (P.Z.); deyuzhijia@163.com (Y.L.)
[2] College of Mechanical and Electronic Engineering, Wuhan Donghu University, Wuhan 430017, China
* Correspondence: liuzhaoxia@wdu.edu.cn (Z.L.); shenzhouxing@taru.edu.cn (H.Z.)

Section I. Experimental Section

Synthesis of MnO NCs@rGO thin-film. All the chemical reagents (analytical grade) were utilized without further treatment. Graphene oxide (0.5−5.0 μm) was purchased from Nanjing XFNANO Materials Tech Co., Ltd. A suspension of $Mn(ac)_2 \cdot 4H_2O$ solution and graphene oxide ($Mn(ac)_2 \cdot 4H_2O$: GO = 10 : 1 mass ratio) was stirred vigorously for 24 hours. An appropriate amount of hydroxylamine (HI, 45%) solution was added and continued stirred for 2 hours. The mixture was then evenly spread on a clean glass plate and dried at 80 ºC for 2 h. Subsequently, the glass plate was submerged into a basin of water, and the brownish black film automatically floated out of the water yielding the flexible MnO_x-rGO intermediate. Finally, the dried MnO_x-rGO intermediates were carbonised in a tube furnace at 500 ºC for 5 h in a mixed atmosphere of 5% H_2/Ar (with a ramp rate of 2 ºC min^{-1}) to finally obtain the flexible MnO NCs@rGO composites.

Material Characterization. The crystalline phase of the samples at various stages was analyzed using X-ray diffraction (XRD, SmartLab9KW, 40 kV, Cu Kα radiation, λ = 0.154 nm) with a scan rate of 20.0 °/min. The morphology and microstructure were characterized by field emission scanning electron microscopy (FESEM, Sirion 200) and

transmission electron microscopy (TEM, Talos F200S, FEI, Thermo). The mass of the active materials was determined through thermogravimetric-differential thermal analysis (TG-DTA) at a heating rate of 10 ºC min^{-1} in flowing air. Elemental composition and valence states were characterized using X-ray photoelectron spectroscopy (XPS, Escalab 250Xi). Raman spectroscopy (Renishaw InVia, 785 nm excitation wavelength) was employed for the analysis of carbon contents.

Electrochemical Measurements. The flexible MnO NCs@rGO and rGO films were cut into discs of ~1 cm^2 diameter as working electrodes. The CR2032 half-cells were assembled with Li-metal, Celgard-2300, and 1.0 M $LiPF_6$ in a mixture of ethylene carbonate/diethyl carbonate (EC/DEC 1:1 by volume) solution as the counter electrode, separator, and electrolyte, respectively. All the coin cells were assembled in an Ar-filled glove box with H_2O and O_2 contents of less than 0.01 ppm. Cyclic voltammetry (CV) and electrochemical impedance spectroscopy (EIS, frequency range from 1.0×10^5 to 0.1 Hz) tests were performed on an Autolab instrument (PGSTAT 302). Galvanostatic charge-discharge tests were performed on a Neware battery tester (Neware CT-4008). The mass loading of the active material of each electrode was ∼1.0 mg cm^{-2}.

The MnO electrodes were prepared by casting a slurry containing active material (80 wt.%), acetylene black (10 wt.%) and polyvinylidene difluoride (PVDF, 10 wt.%) onto Cu foils and then dried for 12 h at 80 ºC in vacuum. The coin-type cells were assembled in an Ar-filled glove box. The electrolyte was a solution of 1.0 M $LiPF_6$ in EC/DEC (1:1 by volume). Using Porous Celgard-2300 as separator and Li metal as counter

electrode.

For the flxible lituium-ion full cell, the binder-free MnO NCs@rGO and $LiCoO_2$ electrodes were used as the anode and the cathode, respectively. The $LiCoO_2$ electrode was fabricated by thoroughly mixing the active material, polyvinylidene difluoride (PVDF), and Super P with a weight ratio of 90:5:5 using N-methyl pyrrolidone (NMP) as a solvent. The slurry was then coated on an aluminum foil and dried in a vacuum oven for 12 h at 80 ºC. The MnO-rGO flexible membrane, $LiCoO_2$ electrode and Celgard-2300 microporous membrane were cut into rectangular shapes with a length and width of 3.0 cm × 4.0 cm, 3.2 cm × 4.2 cm, and 3.4 cm × 4.4 cm, respectively. They were stacked in the order of cathode, separator, and anode, and then packed in an aluminium-plastic film bag. After filling with the appropriate amount of electrolyte (1.0 M $LiPF_6$ in EC/DEC), a soft-packed full battery was assembled under vacuum. Here, long strips of aluminium and copper foil are employed as cathode/anode lugs in the battery, ensuring comprehensive charge transfer in full contact with the corresponding cathode and anode.

Calculation process for the capacitance effect and pseudocapacitive contribution

The capacitance effect can be determined from the CV curves at different scan rates, according to the relationship between measured peak currents (i) and scanning rates (v), as follows:

$$i = a\, v^b, \qquad (1)$$

$$\log i = b \log v + \log a, \qquad (2)$$

where *a* and *b* are the fitting parameters, and *i* and *v* represent peak current and scanning rate, respectively. The variable parameter *b* is deduced from the slope of log (*i*)/log (*v*) within the range of 0.5–1.0. Electrochemical reaction is controlled by ion diffusion if *b* approaches 0.5, while a value near 1.0 means the dominance of capacitive behavior in the electrochemical reaction [1].

Furthermore, the capacitive contribution at various scan rates can be quantitatively calculated via the following equation with the parameters k_1 and k_2:

$$I = k_1 v + k_2 v^{1/2}, \qquad (3)$$

where $k_1 v$ and $k_2 v^{1/2}$ represent the contributions of the capacitive and diffusion behaviors in the reactions, respectively [2].

Calculation process for the diffusion coefficient (D_{Li+})

The galvanostatic intermittent titration technique (GITT) tests were performed by discharging or charging the cells for 30 min at 0.02 A g^{-1}, followed by a 30 min relaxation in the voltage range of 0.01 to 3.0 V. The D_{Li+} can be worked out by solving Fick's second law according to the following equation.

$$D = \frac{4}{\pi\tau}\left(\frac{m_B V_M}{M_B S}\right)^2\left(\frac{\Delta E_S}{\Delta E_\tau}\right)^2 \left(\tau \ll \frac{L^2}{D}\right), \qquad (4)$$

where τ is the relaxation time (s), and m_B, V_M, M_B, and S are the mass, the molar volume of the active material, the molar mass and the area of the electrode, respectively. ΔE_S represents the quasi-thermodynamic equilibrium potential difference between before and after the current pulse. ΔE_τ is the potential difference during the current pulse [3].

Section II. Supplementary Figures and Tables

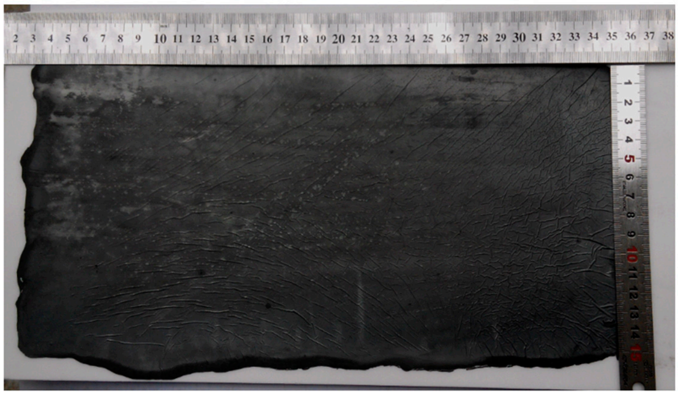

Figure S1. Photograph of the MnO_x-rGO intermediate film.

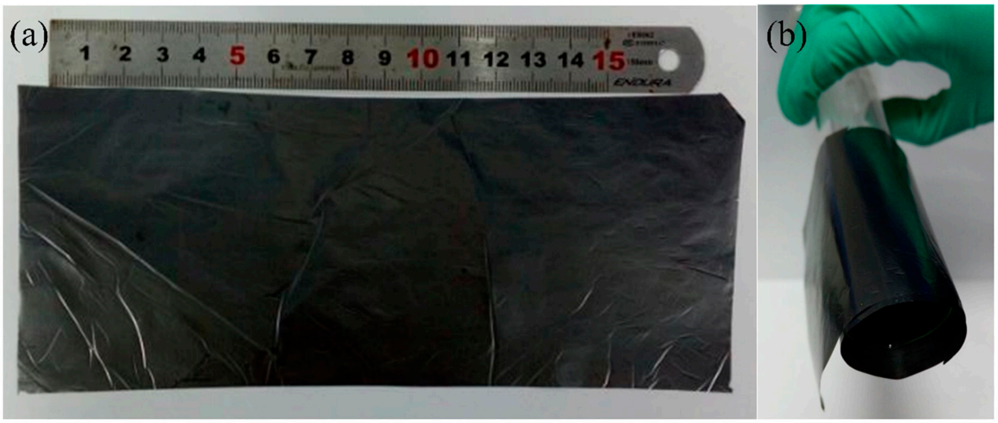

Figure S2. Photograph of the (a) flat and (b) curled MnO NCs@rGO film.

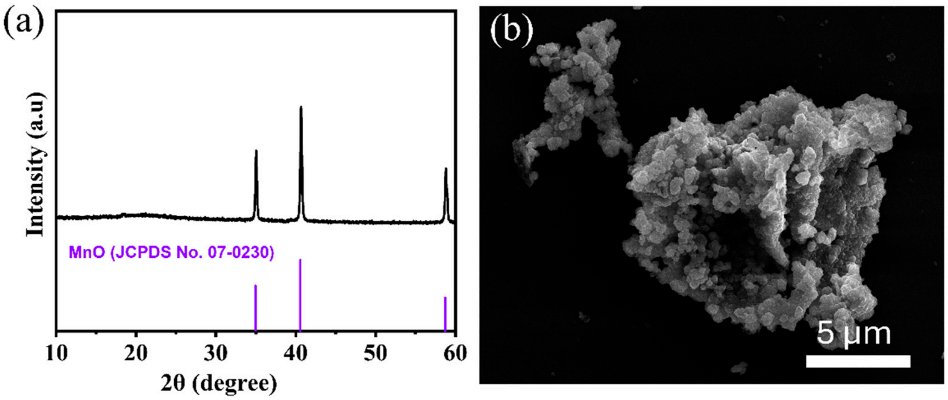

Figure S3. (a) XRD pattern and (b) SEM image of MnO.

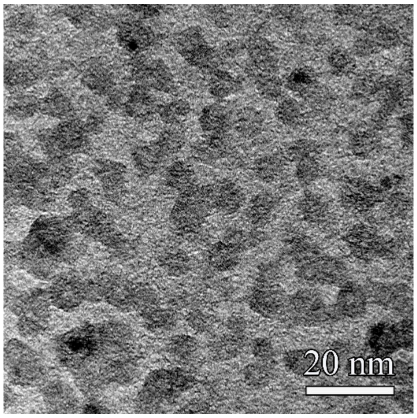

Figure S4. HR-TEM image of the flexible MnO NCs@rGO film.

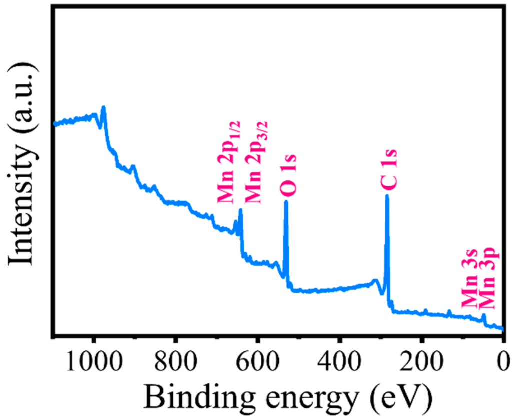

Figure S5.XPS survey spectrum of the flexible MnO NCs@rGO film.

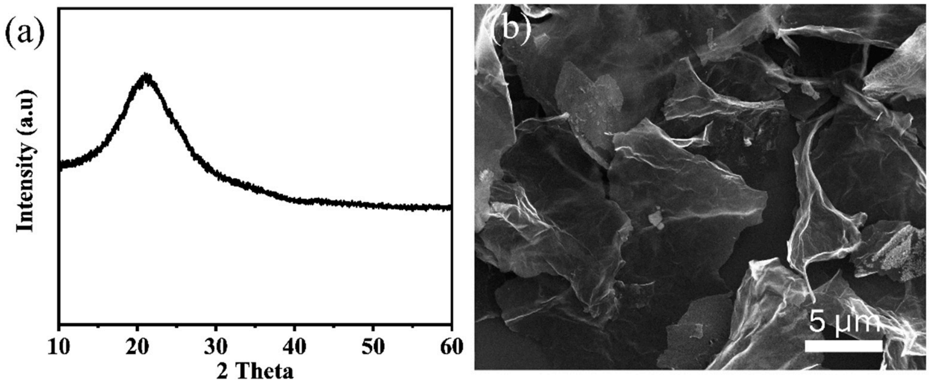

Figure S6. (a) XRD pattern and (b) SEM image of rGO.

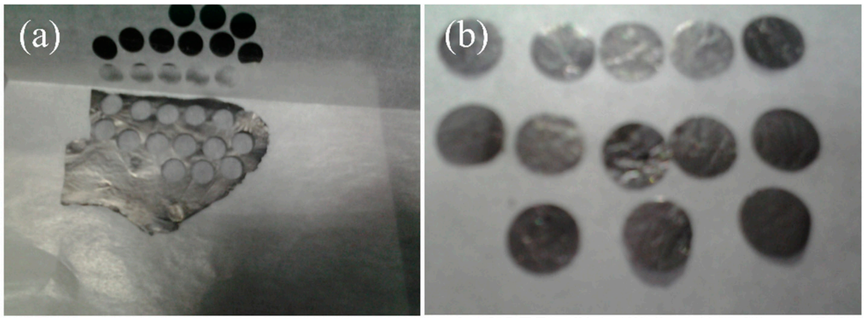

Figure S7. Photograph of the flexible free-standing MnO NCs@rGO electrode.

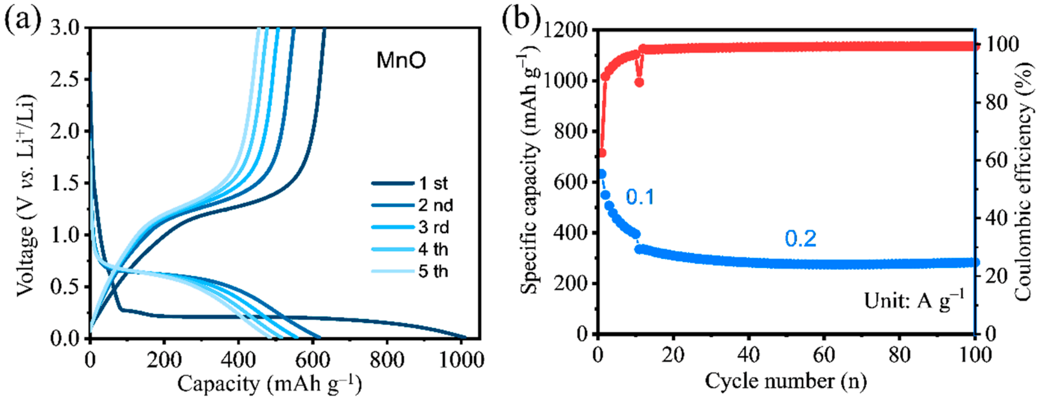

Figure S8. (a) Galvanostatic charge/discharge profiles and (b) cycling performance of MnO anode at 0.2 A g^{-1}.

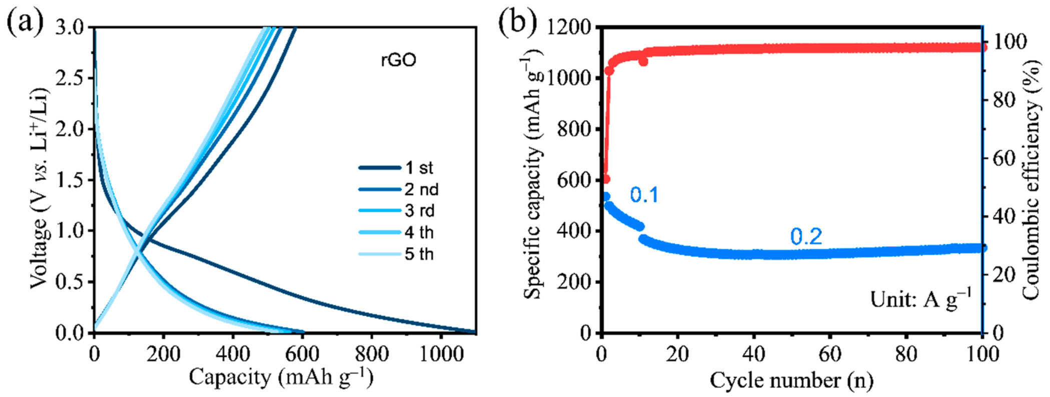

Figure S9. (a) Galvanostatic charge/discharge profiles and (b) cycling performance of rGO anode at 200 mA g^{-1}.

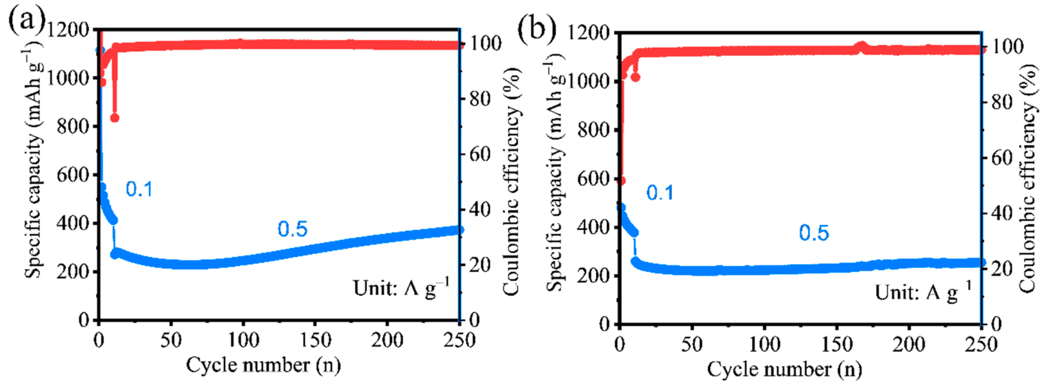

Figure S10. Cycling performance of (a) MnO and (b) rGO anode at 0.5 A g^{-1}.

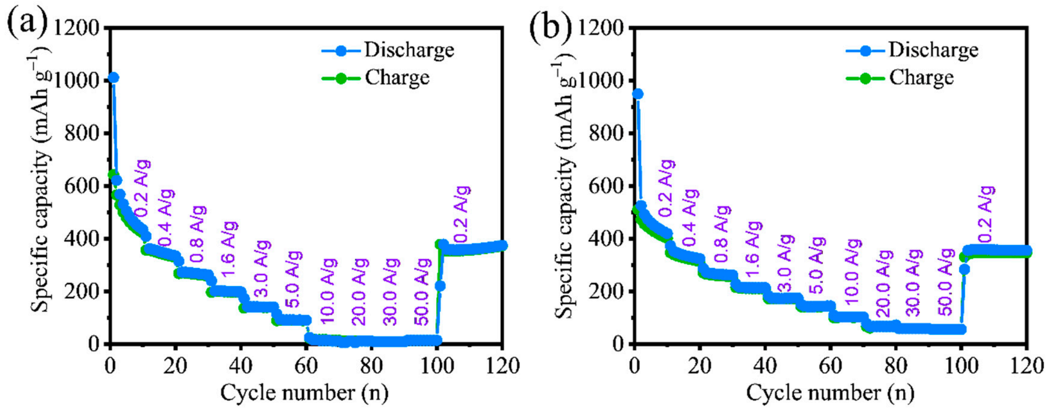

Figure S11. Rate performance of (a) MnO and (b) rGO anode at different current densities from 0.2 A to 50.0 A g^{-1}.

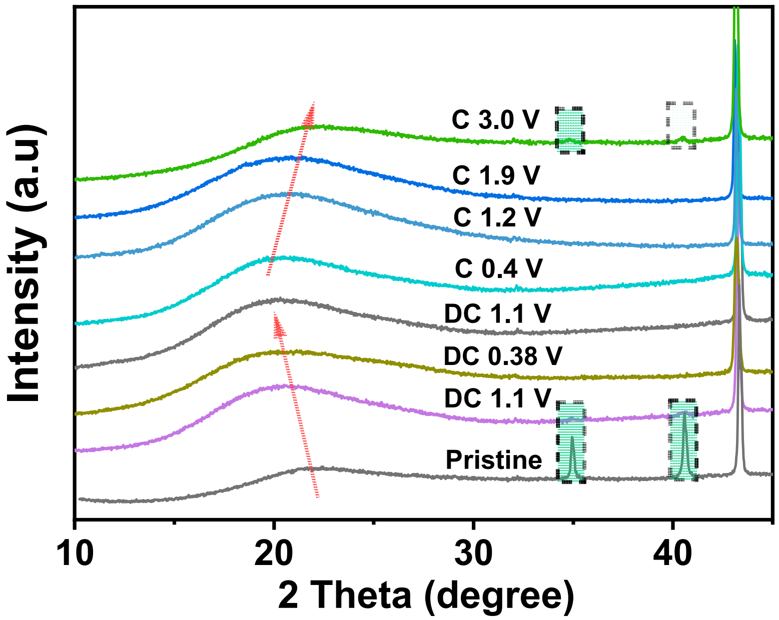

Figure S12. *Ex-situ* XRD results of the flexible MnO NCs@rGO electrode.

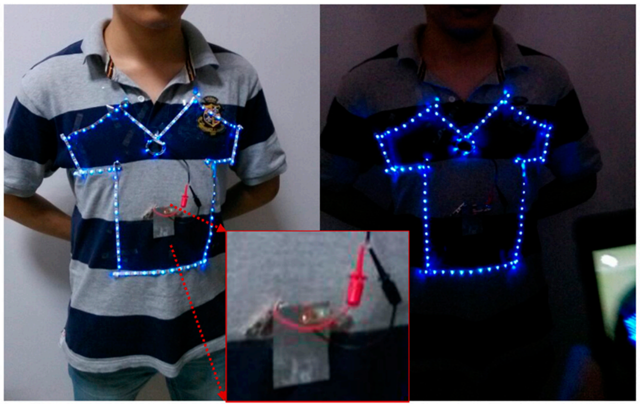

Figure S13. Photograph of the MnO NCs@rGO//$LiCoO_2$ full cell powering an array of 100 LEDs.

Table S1. Comparison of cycling stability between the MnO NCs@rGO anode and other reported MnO_x-based electrodes.

Active materials	Specific capacity	Cyclic stability	Rate performance	Ref.
MnO NCs@rGO	1220 mAh g^{-1}	1000 cycles at 0.5 A g^{-1}	775.4mAh g^{-1}, at 5.0 A g^{-1}<br>667.0 mAh g^{-1}, at 10.0 A g^{-1}<br>515.0 mAh g^{-1}, at 20.0 A g^{-1}<br>336.6 mAh g^{-1}, at 50.0 A g^{-1}	
Mn_2O_3/MnO@NC	683.6 mAh g^{-1}	1000 cycles at 1.0 A g^{-1}	659.2 mAh g^{-1}, at 1.6 A g^{-1}	*J. Colloid. Interf. Sci.* **2024**, *665*, 751
MnO/SCPC	584 mAh g^{-1}	1000 cycles at 1.0 A g^{-1}	615.9 mAh g^{-1}, at 2.0 A g^{-1}	*Chem. Eng. Sci.* **2024**, *285*, 119625
MnO NPs@C Ps	463 mAh g^{-1}	1500 cycles at 1.0 A g^{-1}	*245.8* mAh g^{-1}, at 3.0 A g^{-1}	*J. Phys. Chem. Solids,* **2024**, *187,* 111857
C@MnO QDs/GA	1698 mAh g^{-1}	1000 cycles at 0.2 A g^{-1}	702 mAh g^{-1}, at 2.0 A g^{-1}	*Energy Storage Mater.* **2020**, *27*, 591
3D MnO/C	780 mAh g^{-1}	200 cycles at 2.0A g^{-1}	574 mAh g^{-1}, at 2.0 A g^{-1}	*J. Alloy. Compd.* **2023**, *948*, 169799;
F-MnO@FG	382.2 mAh g^{-1}	2000 cycles at 5.0 A g^{-1}	316.9 mAh g^{-1}, at 5.0 A g^{-1}	*J. Alloy. Compd.* **2023**, *945*, 169255
$ZnMn_2O_4$	811 mAh g^{-1}	100 cycles at 0.2 A g^{-1}	811 mAh g^{-1}, at 3.2 A g^{-1}	*J. Colloid. Interf. Sci.* **2023**, *641.* 386
Mn_3O_4/biochar	895 mAh g^{-1}	200 cycles at 0.5 A g^{-1}	385.8 mAh g^{-1}, at 1.6 A g^{-1}	*J. Colloid. Interf. Sci.* **2024**, *669.* 740
Fe-MnO@C	512.3 mAh g^{-1}	400 cycles at 1.0A g^{-1}	394.2 mAh g^{-1}, at 3.0 A g^{-1}	*J. Alloy. Compd.* **2024**, *982*, 173805

REFERENCES

1. Li Q.H.; Yu, D.D.; Peng, J.; Zhang, W.; Huang, J.L.; Liang, Z.X.; Wang, G.Y.; Li, H.X.; Xiong, S.Y.; Wang, J.Z.; Huang, S. M. Efficient Polytelluride Anchoring for Ultralong-Life Potassium Storage: Combined Physical Barrier and Chemisorption in Nanogrid-in-Nanofiber, *Nano-Micro Letters.* **2024**, *16*, 77.

2. Peng, B.; Xu, S.; Lv, Z.; Zhang, S.; Gao, Y.; Lin, T.; Huang, F. Toward Extremely Fast Charging Through Boosting Intercalative Redox Pseudocapacitance: A $SbCrSe_3$ Anode for Large and Fast Sodium Storage. *Adv. Energy Mater.* **2022**, *13*, 2203187.

3. Dong, C.; Shao, H.; Zhou, Y.; Du, W.; Li, L.; Sun, J.; Yan, Z.; Hu, Z.; Chou, S.; Jiang, F. Construction of ZnS/Sb_2S_3 Heterojunction as an Ion-Transport Booster toward HighPerformance Sodium Storage. *Adv. Funct. Mater.* **2022**, *33*, 2211864.
